# From Postpartum Haemorrhage Guideline to Local Protocol: A Study of Protocol Quality

**DOI:** 10.1007/s10995-016-2050-9

**Published:** 2016-07-09

**Authors:** Mallory D. Woiski, Helena C. van Vugt, Anneke Dijkman, Richard P. Grol, Abraham Marcus, Johanna M. Middeldorp, Ben W. Mol, Femke Mols, Martijn A. Oudijk, Martina Porath, Hubertina J. Scheepers, Rosella P. Hermens

**Affiliations:** 1 Department of Obstetrics and Gynaecology, Radboud University Medical Centre, Nijmegen, Geert Grootplein 10, P.O. Box 9101, 6500 HB Nijmegen, The Netherlands; 2 Department of Obstetrics and Gynaecology, Reinier de Graaf Hospital, P.O. Box 5011, 2600 GA Delft, The Netherlands; 3 Institute for Quality of Healthcare (IQ Healthcare), Radboud University Nijmegen Medical Centre, P.O. Box 9101, 6500 HB Nijmegen, The Netherlands; 4 Department of Anesthesiology, Maastricht University Medical Centre, P.O. Box 5800, 6202 AZ Maastricht, The Netherlands; 5 Department of Obstetrics, Leiden University Medical Centre, P.O. Box 9600, 2300 RC Leiden, The Netherlands; 6 Department of Obstetrics and Gynaecology, Academic Medical Centre (AMC), P.O. Box 22660, 1100 DD Amsterdam, The Netherlands; 7 Department of Obstetrics and Gynaecology, University Medical Centre Utrecht, P.O. Box 85500, 3508 GA Utrecht, The Netherlands; 8 Department of Obstetrics and Gynaecology, Maxima Medical Centre, P.O. Box 90052, 5600 PD Veldhoven, The Netherlands; 9 Department of Obstetrics and Gynaecology, Maastricht University Medical Centre, P.O. Box 5800, 6202 AZ Maastricht, The Netherlands

**Keywords:** Health care quality access and evaluation, Guideline adherence, Postpartum hemorrhage, Clinical protocols

## Abstract

*Objective* Postpartum hemorrhage (PPH) has a continuously rising incidence worldwide, suggesting suboptimal care. An important step in optimizing care is the translation of evidence-based guidelines into comprehensive hospital protocols. However, knowledge about the quality of these protocols is lacking. The objective of this study was to evaluate the quality of PPH-protocols on structure and content in the Netherlands. *Methods* We performed an observational multicenter study. Eighteen PPH-protocols from 3 University Hospitals (UH), 8 Teaching Hospitals (TH) and 7 Non-Teaching hospitals (NTH) throughout the Netherlands were acquired. The structure of the PPH-protocols was assessed using the Appraisal of Guidelines for Research and Evaluation (AGREE-II) Instrument. The content was appraised using previously developed quality indicators, based on international guidelines and Advance-Trauma-Life-Support (ATLS)-based course instructions. *Results* The quality of the protocols for postpartum hemorrhage for both structure and content varied widely between different hospitals, but all of them showed room for improvement. The protocols scored mainly below average on the different items of the AGREE-II instrument (8 of the 10 items scored <4 on a 1–7 scale). Regarding the content, adoption of guideline recommendations in protocols was 46 %. In addition, a timely indication of ‘when to perform’ a recommendation was lacking in three-fourths of the items. *Conclusion* This study shows that the quality of the PPH-protocols for both structure and content in the Netherlands is suboptimal. This makes adherence to the guideline and ATLS-based course instructions difficult.

## Significance

Postpartum hemorrhage (PPH) remains a major problem in high resource countries, regardless of the development and dissemination of evidence-based clinical guidelines including the instructions on Advance Trauma Life Support (ATLS)-based courses for obstetric emergencies. Putting evidence-based PPH recommendations into practice begins with the translation of evidence-based guidelines into high quality local protocols. For many care providers these protocols often are the only guide in the prevention and management of PPH in the actual care. However a recent study (Bialit et al. AJOG 2015) showed that merely the presence of PPH-protocols does not indicate a better outcome. Variation in the quality of these protocols could be a possible explanation. This quality and its variation both regarding structure and content is yet unknown. This manuscript gives insight in an underexposed but in our opinion very important component of PPH-care and shows room for improvement. This manuscript not only concerns obstetricians but any professional in any country working with such guidelines. Therefore we think this manuscript fits perfectly in the scope of the Maternal and Child Health Journal.

## Introduction

Postpartum Hemorrhage (PPH) is the number one cause of worldwide maternal death [21]. It does not only have its origin in low resource countries, but developed countries also contribute [13, 9, 22, 32]. A high proportion (72–90 %) of the morbidity of obstetric hemorrhage is considered to be preventable if adequately managed through early recognition, adequate interventions in early stages and proper choices of therapies [8, 12, 5]. Actually, PPH-care consists of a prevention phase and a treatment phase, where different actions must be taken by different professionals, consecutively or simultaneously, in a limited time-frame, for PPH can develop into an urgent life-threatening situation that requires an immediate response [20].

Evidence-based guidelines can assist professionals in standardizing adequate management and support the clinical evidence-based decision making [17]. Advanced Trauma Life Support (ATLS) courses educate the professionals in using a highly structured multidisciplinary approach of obstetric emergencies such as PPH [20]. Streamlining day-to-day PPH-care for every professional on the basis of evidence-based PPH-guidelines and ATLS-based course instructions is a challenge [17]. Several national societies of maternal-fetal-medicine [18, 15] strongly recommend the use of protocols as a way to streamline PPH-care, because compliance of guidelines improves if a protocol is present [11, 24, 26]. In fact, for the majority of the professionals, such as nurses, midwives and residents, these protocols are the main guide in the prevention and management of PPH. However, a recent study showed that merely the presence of PPH-protocols does not mean a better outcome [4]. Variation in the quality of these protocols could be a possible explanation. This quality and its variation, both regarding structure and content, is yet unknown. Therefore, we aimed to evaluate the quality of PPH-protocols, both on structure and content, in the Netherlands.

## Materials and Methods

### Design, Setting and Study Population

We performed an observational multicenter study. The study was established within the Dutch Consortium for Healthcare Evaluation in Obstetrics and Gynecology. This Consortium aims at extending evidence-based medicine in obstetrics and improving the quality of the Dutch obstetric care. Nowadays all ten Dutch Perinatology Centers participate in this Consortium, together with 70 Dutch general hospitals. A viable selection of 1:5 of the Dutch hospitals was made and a total of eighteen (23 %) PPH-protocols from these Dutch hospitals that provide acute obstetric care were collected from February 2011 through February 2012. The selection of hospitals was based on the different types of hospitals [University Hospitals (UH), Teaching Hospitals (TH) and Non-Teaching Hospitals (NTH)], with a similar distribution by type across the country (3 UH, 8 TH, 7 NTH). The obstetrician of these hospitals was contacted through e-mail or telephone with the question to send us a copy of their most recent local PPH protocol, and all the hospitals willingly provided us with a copy.

### Assessment of Protocol Quality

To evaluate the quality of the included protocols on structure and form, we used the Appraisal of Guidelines for Research & Evaluation (AGREE-II) instrument [2]. This instrument offers a systematic framework for assessing the most important aspects of quality of guidelines. We selected the following 10 from 23 scoring items for assessing form and structure of the protocols: objective, title with health questions and patient population (domain Scope and Purpose), publication date, revision date, externally reviewed yes/no and references (domain Rigor of Development), authors and target group (domain Stakeholder Involvement) and use of appendices/tools (domain Applicability). The remaining 13 items of the AGREE-II instrument were rated as unsuitable for assessing the protocols because they particularly relate to the process used to gather and synthesize evidence, cost implications and editorial independence. The AGREE-II-items were scored on a 7-point scale from “totally disagree” to “totally agree” (score 1–7).

To evaluate the quality of the included protocols on content according to PPH-guidelines and the ATLS-based course, we used guideline-based quality indicators for prevention (n = 2), management (n = 15) and organization (n = 5) of PPH (Table 1) [30]. These indicators were previously developed according to the RAND-modified Delphi method to measure guideline adherence in the actual care, and are based on different international PPH-guidelines, including the guidelines from the World Health Organization (WHO), international literature and ATLS-based courses [20, 15, 3, 28, 31]. The indicators for management of PPH were classified into three subsequent stages of seriousness of PPH, in terms of the amount of blood loss and/or signs of shock, namely: 1. >500 mL, 2. >1000 mL or >500 mL with signs of shock, and 3. >2000 mL. This set can be used to measure the actual performances and whether the performances are carried out in the right stages of blood loss.

**Table 1:** Guideline-based quality indicators for prevention, management and organization of PPH-care

Performance indicators for prevention of PPH	
Prevention	To identify patients at high risk of PPH during pregnancy at the out-clinic and during labor, determine or adapt a policy for parturition and document it To ensure IV access during labor, provide an active management of the third stage of labor and objectively measure blood loss
Performance indicators for management of PPH: In case of a patient with PPH the clinician should*…*	
*Time*	
Communication documentation	
>500 mL	Inform the gynecologist (in training)
>1000 mL	Call for the obstetrician on ward (if the clinician is not a gynecologist), the anesthetist and surgery personnel, and transport patient to the operating room if the bleeding persists Allocate one member of the team to record vital signs, events, fluids, and drugs
>2000 mL	Call for a second obstetrician and inform the radiologist (if applicable)
Monitoring and prevention of shock	
>500 mL	Monitor vital functions appropriately, take blood samples and replace fluid Continuously monitor pulse and oxygen saturation and BP (5–10 min) Take blood samples: FBC and cross match screen Ensure an IV access (18 gauge) and commence volume replacement (1 l of saline)
>1000 mL	Monitor additional vital functions appropriately, give oxygen and replace fluid Give 10–15 L/min oxygen through face mask regardless of her oxygen saturation Monitor urine production Provide a second IV access (18 gauge), and replace volume by using pressure bags and warmed fluid (in case of large volumes)
>2000 mL	Call for anesthetic assistance if the airway is compromised
Blood products	
>1000 mL	Urgently order units of blood and fresh frozen plasma, check and correct clothing status
>2000 mL	Follow hospital-wide mass transfusion protocol Transfuse uncrossed matched O negative blood if hemorrhage is life threatening, correct clothing status *including platelets* >*50 or when surgery is planned* >*80*
Therapy	
>500 mL	Treat uterine atony Continuous uterus massage, bladder catheterization and uterotonic medication in steps In case of retained placenta: perform controlled cord traction followed by placenta removal in the operating room
>1000 mL	Treat PPH as an atony till proven otherwise, use prostaglandins IV if other uterotonic treatment fails
>2000 mL	Perform or consider following interventions (Perform) empty uterus, repair genital tract injury (vaginal, cervical uterine rupture) (Consider) selective arterial embolization as alternative or in addition to surgical intervention, if not successful consider internal iliac artery balloon (Consider) Brace suture, arterial ligation and hysterectomy In an emergency situation to temporarily stop bleeding and catch up resuscitation, organize the next intervention or transport patient to a tertiary centre: - perform: bimanual compression of the uterus, aorta compression and place Bakri balloon or uterine tamponade through packing (also therapeutically)
Organizational indicators for PPH: *In every hospital system…*	
Protocols and agreements	The following local protocols and agreement should be available Protocol PPH according to the national guideline Local mass transfusion protocol Protocol for women refusing blood products A written agreement between the related disciplines (anesthesia, hematology, radiology) for a multidisciplinary approach in the treatment of PPH
Accessibility	It must be clear how to rapidly reach the following staff/departments at any moment 1. Gynecologist; 2. Anesthesiologist; 3. Hematologist; 4. Intensive care specialist; 5. Surgery team; 6. Blood bank; and 7. Resuscitation team There should be clear prior agreements about the time interval between the call and availability of the following staff (gynecologist, anesthesiologist and surgery team)
Audit and feedback	PPH cases should be Discussed during morning team-gathering in a structured and detailed way, according to local PPH-protocol/guideline Monitored by multidisciplinary audit and/or confidential enquiries on a regular basis with the associated caregivers, to identify problems that need reorganization and or training
Documentation and registration	The practitioner must ensure proper documentation for each PPH case, in particular concerning the time course All cases of PPH (>1000 cc) must be registered

Published in Woiski et al. [30]: Guideline-based development of quality indicators for prevention and management of postpartum hemorrhage

Twenty (from 22) indicators were relevant to assess the content of protocols and we transformed them into 92 measurable items. All protocols were scored on the presence or absence of these items. In addition, items regarding ‘actions in the management of PPH’ were evaluated on whether they were accompanied by a description of ‘when’ (in terms of the amount of blood loss or vital signs) these actions would have to be taken. For example; it is recommended to place a second drip in the event of more than 1000 mL blood loss.

Two independent researchers performed all measurements.

### Statistical Analysis

With regard to the structure of the protocols we calculated median scores per AGREE-II domain, for all the hospitals together and per type of hospital. The results regarding content were analyzed descriptively. At first a total score was calculated, meaning the sum of all present items (Y) in the 18 hospital protocols divided by the maximum amount of items (Y/92 X 18). Subsequently, frequencies per item per type of hospital were assessed. Cohen’s kappa was calculated to measure conformity between the two assessors (HvV and FM) and totaled 0.9 for both structure and content measurements. All measurements were analyzed using SPSS 20,0.

## Results

The quality of the analyzed protocols differed substantially for both structure and content.

Regarding the structure of the protocols the length of the total protocol varied, for example from half a page to five pages, and the presence of headlines and paragraphs varied, as well as the presence or absence of a flowchart. The presence of appendices/tools in the domain “Applicability” had a median score of 3 [ranging from 2 (TH and NTH) to 3 (UH)] (Table 2).

**Table 2:** Quality of the protocols on structure using the AGREE-II instrument

AGREE-II domain	Form and structure		Total (Median) (range 1–7) n = 18	UH (Median) (range 1–7) n = 3	TH (Median) (range 1–7) n = 8	NTH (Median) (range 1–7) n = 7
Scope and purpose	1	Objective	1	7	1	7
	2	Title with health question	7	7	7	7
	3	Patient population	2	2	2	2
Rigor of development	4	Publication date	5	5	3	7
	5	Revision date	1	1	1	1
	6	Externally reviewed	1	1	1	1
	7	References	1	4	1	1
Stakeholder	8	Authors	2	2	1	7
Involvement	9	Target group	2	3	2	1
Applicability	10	Appendices/tools	3	3	2	2

*UH* University Hospitals, *TH* Teaching Hospitals, *NTH* Non Teaching Hospitals

With respect to the domain “Scope and Purpose” a clear title with health question was found in all protocols (median score of 7), unlike the item ‘patient population’, which was predominantly absent (median score of 2). Items in the domains “Stakeholder Involvement” and “Rigor of Development” did not score well in almost all protocols, in particular those from the TH. From all these items, the item ‘publication date’ scored best [median score of 5, ranging from 3 (TH) to 7 (NT)].

Overall, the scores on the different items on the AGREE-II instrument were mostly below average, e.g. eight of the total of ten items scored below four on a scale of one to seven.

Regarding the content of protocols about half (46 %) of the total number of 92 items could be found in 18 protocols ranging from 20 % in a NTH to 68 % in a UH (Table 3). Below we present the main results for the different stages of PPH-care.

**Table 3:** Quality of local protocols on content

Items	Total (n = 18) %	UH (n = 3) %	TH (n = 8) %	NTH (n = 7) %
Overall mean score of the items in the protocols (range)	46 (20–65)	55 (50–65)	48 (35–64)	39 (20–54)
Prevention of PPH				
Identification and determining policy of patients at high-risk for PPH				
At outpatient clinic	11	0	25	0
During labor	33	33	38	29
Active management of the third stage of labor	22	33	0	43
Objectify (weigh) blood loss of high-risk patients	67	33	63	86
Management PPH >500 mL				
Call for the gynaecologist on ward	72	67	88	57
Continuously monitor heart rate	6	0	0	14
Continuously monitor oxygen saturation	11	0	13	14
Measure blood pressure (5–10 min)	28	33	13	43
Ensure drip	94	100	100	86
Assess cross match blood	100	100	100	100
Assess hemoglobin	94	100	88	100
Continuous uterus massage	78	100	63	86
Bladder catheterization	100	100	100	100
To give uterotonic medication in steps	94	100	88	71
Medication plan in steps present in protocol	88	100	88	86
If retained placenta, remove placenta in operating room	72	100	75	57
>1000 mL				
Give 10–15 l of oxygen through face mask	56	67	75	29
Order packed cells	94	100	100	86
Provide a second drip	88	100	100	71
Monitor urine production	56	100	75	14
Control and correct blood clotting	78	100	75	71
Allocate one member of the team to record events	17	33	13	14
Call for the anaesthesiologist on ward	6	33	0	0
Call for the operating team on ward	11	33	13	0
Replace volume by using pressure bags	33	67	25	29
>2000 mL				
Transfuse uncrossed matched O negative blood if PPH is life threatening	11	0	13	14
Follow the local shock protocol	6	0	13	0
Call for a second gynecologist/perinatologist	17	33	13	14
Consider embolization [if embolization possibility is present in the hospital (n = 17)]	70	100	88	33
Consider brace suture	50	33	63	43
Consider a timely hysterectomy	6	33	0	0

*UH* university hospitals, *TH* teaching hospitals, *NTH* Non teaching hospitals

### Prevention of PPH

Recommendations concerning identification of high-risk patients during labor were found in 33 % [ranging from 29 % (NTH) to 38 % (TH)] of the protocols. Active management of the third stage recommendation was included in 22 % of the protocols [ranging from 0 % (TH) to 43 % (NTH)].

### Management of PPH

Recommendations for continuous monitoring the vital parameters, e.g. pulse, O2-saturation and blood pressure, were included respectively in 6 % [ranging from 0 % (UH and TH) to14 % (NTH)], 11 % [ranging from 0 % (UH) to 14 % (NTH)] and 28 % [ranging from 13 % (TH) to 43 % (NTH)] of the total protocols. In all protocols was stipulated that cross-match blood has to be taken and in almost all protocols, except for one TH, that packed cells should be ordered. However, 11 % of the protocols mentioned to in a serious situation give O-negative blood in the absence of cross-match blood, with a range of 0 % in the UH to 14 % in the NTH. Half of the protocols suggest to consider a B-Lynch suture (33 % UH, 43 % NTH and 63 % TH), however, to consider a timely hysterectomy was found in only one protocol [6 % (UH)].

### Time Factor

**Fig. 1:**
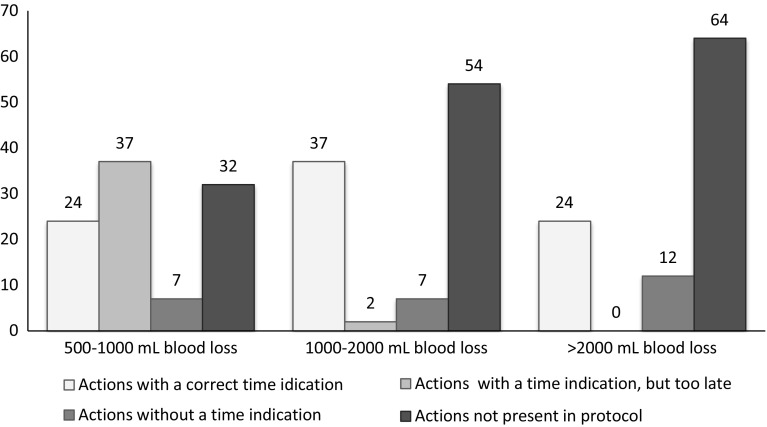
Mean percentage of items with a time indication in the protocols

Of 92 items, 61 indicated at what stage (expressed in the amount of blood loss or shock signs) action should be taken. Of the items that should be performed at the stage of 500–1000 mL blood loss, only 24 % gave an indication of when or under which circumstances these had to be undertaken (Fig. 1). Unfortunately, 76 % of these items were either mentioned with an incorrect time indication (too much blood loss) or without any time indication, or were not mentioned in protocols at all. This also counts for the next stages (1000–2000 mL and >2000 mL blood loss), where 63 and 76 % of the items had an incorrect time indication, no time indication, or could not be found in the protocols at all.

## Discussion

This study shows a large variation between hospitals in the quality of protocols for postpartum hemorrhage as regards both structure and content. The protocols scored mostly below average on the different items of the AGREE-II domains [8 out of 10 items scored below 4 (1–7 scale)]; protocols of the TH in particular scored lower (9 out of 10 items scored below average) compared with the UH and NTH (6 out of 10 items scored below average).

Regarding the content, less than half (46 %) of the total number of 92 items were found in the 18 PPH-protocols. The content of the protocols of the NTH was the least in accordance with the guideline and ATLS-based course instructions (33 % NTH, 48 % TH, 55 % UH). Furthermore, as regards items that needed a time indication on ‘when to perform’, about three-fourths of these items were mentioned with either an incorrect time indication (too much blood loss), no time indication at all, or were simply not present in the protocols. So, the overall quality of protocols showed much room for improvement.

A limitation of this study is, that we did not relate the quality of the protocols to the compliance with the guidelines in the actual care. It is possible that the current care is more in accordance with the guidelines than we now assume based on the protocols. It is possible that the variation in quality found could explain the findings of Bailit et al. [4] that the presence of protocols does not improve care as a rule. Therefore, to measure the current care will be the next step. A second limitation is that we used the Agree-II instrument which is meant to be used for Guidelines. However, a quality instrument for local protocols does not exist, and because local protocols are based on guidelines, the Agree-II instrument is the best instrument which met this purpose.

The strength of our study, however, is that we investigated the quality of local PPH-protocols, including both structure and content. Until now, the few studies that were performed regarding protocols only concerned the presence or absence of protocols, not the quality thereof (see below).

Specific omission of highly relevant clinical items could lead to a delayed recognition and treatment of PPH by the immediate care providers using the protocols. In our study, highly relevant clinical items in the prevention of PPH, such as identifying a high-risk patient, active management of the third stage and monitoring blood loss in high-risk patients, were only present in respectively 11, 22 and 67 % of the protocols. Furthermore, in the management of PPH, to monitor vital signs in case of a PPH was only found in less than one-third of the protocols. Delay and denial are key contributors to poor outcome in PPH while prevention and early recognition of PPH provide better results [8, 12, 5, 18, 6, 25]. A risk assessment of the outpatient-clinic patients, which helps identify high-risk patients, will increase vigilance of the staff and the taking of extra precautions when necessary. An active management of the third stage, as is strongly supported by evidence, diminishes blood loss [6, 27]. Moreover, proper management of PPH includes analyzing maternal status for early recognition through accurate estimation of blood loss, vital signs monitoring and prompt intervention in the early stages using a rapid and adequate multifaceted approach [12, 18]. Different international guidelines highlight the evaluation of vital signs and recommend more accurate management for PPH if blood loss causes changes in vital signs [15, 28, 7]. Omission of these items in protocols may be a factor for improper management of PPH; in our study only one out of 18 protocols suggested to monitor the pulse rate continuously if a PPH occurs. Certainly, it is arguable that these factors are a part of common knowledge and practice, but, the direct care providers in the PPH-care and therefore the ones who are responsible for the prevention and early recognition of PPH are usually the ones with the least experience, especially in the TH. Besides, midwives and nurses who are the professionals primarily responsible for ensuring patient safety, work mainly protocol based and use these protocols as their written source of knowledge and guidance in the daily care [26]. Therefore, it has to be clear for the direct care providers dealing with PPH, which acts must be performed at what amount of blood loss and at what condition the patient in. Unfortunately, only one-third of the items with a time indication were correctly described in the protocols.

In literature little is found about the incorporation of guideline-recommendations in protocols. Cromwell et al. [14] shows that only 20 % of all protocols in Great-Britain took over all recommendations of the national guideline “Group B Streptococcus”. In our study, we see a similar trend. Lack of familiarity with a guideline’s content, with the relevant research literature, disagreement with the guideline’s interpretation of the literature, but also the ways in which recommendations are formulated are reported factors for not adopting guideline-recommendations [17, 16]. Cameron et al. [10] investigated current Australian practice in the development of a local policy with regard to prevention, early recognition and management of PPH. They found time and staffing issues to be significant barriers to local policy development from guidelines, especially the deficiency of skills and experience needed to develop written protocols.

It is known that not only the content is important for delivering proper care, but the protocol must be feasible and have a clear structure for the direct caregivers as well [19]. Improved compliance with protocols is found if there are comprehensive protocols, especially if nurses are involved in the development of these protocols [1]. In our study the structure of the studied protocols differed greatly whereas the TH scored lower than the other two types of hospitals.

In order to improve adoption of guideline-recommendations and not to ‘keep reinventing the wheel’, guidelines should come up with a template or model protocol with a clear format, better structuring and with all the important guideline-recommendations that can easily be adapted to the local situation [10, 19]. This template could be accompanied by additional materials such as a summary document, flowcharts educational tools, patient leaflets, or computer support for improving compliance with the protocols and therefore the guideline. It is known that the WHO has presented the recommendation as a list to be followed in case of a PPH and the FIGO has prepared a prevention and management protocol for PPH [29, 23]. Despite the fact that these two guidelines mainly focus on low resource countries, they could be adopted by other countries as well.

## Conclusion

This study shows that the quality of the local PPH-protocols for both structure and content is suboptimal, especially the adoption of guideline-recommendations in protocols. This makes adherence to the guideline and ATLS-based course instructions difficult. It is possible, however, that the current care is more in accordance with the guideline than we now assume based on the protocols. Therefore, to measure the current care will be the next step. In the future more attention and assistance is needed to ensure the quality of protocols, for example by adding a standard protocol template, flowcharts and checklists to PPH-guidelines.
